# Pilot Study of Circulating Tumor Cells in Early-Stage and Metastatic Uveal Melanoma

**DOI:** 10.3390/cancers11060856

**Published:** 2019-06-20

**Authors:** Kartik Anand, Jason Roszik, Dan Gombos, Joshua Upshaw, Vanessa Sarli, Salyna Meas, Anthony Lucci, Carolyn Hall, Sapna Patel

**Affiliations:** 1 Houston Methodist Cancer Center, Houston, TX 77030, USA; kartikanand88@gmail.com; 2 Department of Melanoma Medical Oncology, UT MD Anderson Cancer Center, Houston, TX 77030, USA; jroszik@mdanderson.org; 3 Department of Head and Neck Surgery, Section of Ophthalmology, UT MD Anderson Cancer Center, Houston, TX 77030, USA; dgombos@mdanderson.org; 4 Department of Surgical Oncology, UT MD Anderson Cancer Center, Houston, TX 77030, USA; jrupshaw@mdanderson.org (J.U.); vnsarli@mdanderson.org (V.S.); smeas@mdanderson.org (S.M.); alucci@mdanderson.org (A.L.); cshall@mdanderson.org (C.H.)

**Keywords:** uveal melanoma, liquid biopsy, circulating tumor cells, pilot study

## Abstract

Nearly 50% of uveal melanoma (UM) patients develop metastatic disease, and there remains no current standard assay for detection of minimal residual disease. We conducted a pilot study to check the feasibility of circulating tumor cell (CTC) detection in UM. We enrolled 40 patients with early or metastatic UM of which 20 patients had early-stage disease, 19 had metastatic disease, and one was not evaluable. At initial blood draw, 36% of patients had detectable CTCs (30% in early-stage vs. 42% in metastatic), which increased to 54% at data cutoff (40% in early-stage vs. 68% in metastatic). Five early-stage patients developed distant metastases, 60% (3/5) had detectable CTCs before radiographic detection of the metastasis. Landmark overall survival (from study enrollment) at 24 months was statistically lower in CTC-positive vs. negative early-stage UM (*p* < 0.05). Within this small dataset, the presence of CTCs in early-stage UM predicted an increased risk of metastatic disease and was associated with worse outcomes.

## 1. Introduction

Uveal melanoma, the most common primary intra-ocular malignancy, is a rare neoplasm that constitutes less than 5% of total melanoma incidence [1]. The most common site involved is the choroid (90%), followed by the ciliary body (6%) and the iris (4%) [2]. In the U.S., the age-adjusted risk of uveal melanoma is 5.1 per million [3]. Caucasians are at increased risk of uveal melanoma and the mean age of diagnosis is 62 years [4]. Risk factors include fair skin, blonde hair, and light eye color, presence of choroidal nevus and presence of germline breast cancer 1-associated protein 1 (BAP1) mutation. The role of ultraviolet exposure as a risk factor remains controversial [5]. Less than 5% of the cases at initial ocular presentation have distant metastasis. Primary tumor management generally involves globe sparing local therapy such as laser or radiation; alternatively, enucleation is an option. Prognostic factors to help predict the risk of metastasis include cytogenetics, gene expression profiling by RNA-based techniques and mutational analysis [6]. Increased expression of ABCB5 protein is also linked to poor prognosis and increased risk of metastasis [7]. It is common practice to have surveillance scans with abdominal imaging (CT or MRI) every 3 to 6 months post primary tumor management [6]. As approximately half of patients with uveal melanoma develop metastatic disease, there is need to enhance our ability to detect minimal residual disease (MRD). A “liquid biopsy” approach, based on the assessment of rare circulating tumor cells (CTCs) or DNA from peripheral blood samples, provides a promising method to permit identification of early recurrence or to monitor disease status. Liquid biopsies are easily obtained, minimally-invasive, longitudinal snapshots that can be used to measure micro-metastatic disease burden, monitor disease progression, and provide real-time genomic assessments of primary tumor/metastatic lesions. As hematogenous spread remains the route of dissemination in uveal melanoma, there is particular interest in the role of CTCs. CTCs have been shown to initiate metastasis in pre-clinical mouse xenograft models [8]. The use of CTCs as a prognostic marker has been evaluated in numerous solid organ malignancies including breast, prostate, colon, and bladder and esophageal cancer where high CTC count is correlated with increased risk of metastasis [9]. We designed a study to evaluate the role of CTCs in uveal melanoma in patients presenting to the University of Texas MD Anderson Cancer Center, Melanoma and Skin Center. Our main objective was to check the feasibility of CTC detection in uveal melanoma. Our secondary objectives were to evaluate if CTCs varied with risk status in early-stage uveal melanoma and if the presence of CTCs predicted risk of distant metastasis.

## 2. Materials and Methods

### 2.1. Patients

We enrolled 40 patients who presented to the University of Texas MD Anderson Cancer Center Uveal Melanoma medical oncology clinic with early-stage or metastatic uveal melanoma from 1 December 2014 to 1 February 2018 in an IRB-approved study (LAB11-0314). All patients provided written, informed consent and were 18 years of age or older. The demographic information collected included age, sex and race, date of diagnosis of primary uveal melanoma or metastasis, and dates of CTC collection. Data from 39 patients was available for evaluation. 

### 2.2. Risk Stratification

Primary uveal melanoma patients were risk-stratified based on the commercially available DecisionDX-UM assay, which uses RT-PCR to determine the gene expression of 15 genes in the tumor sample. The assay is validated to determine the risk of distant metastasis and stratifies early-stage uveal melanoma based on increasing risk of 5-year distant metastasis into Class 1A, 1B and 2 with Class 1 tumors considered lowest-risk and Class 2 tumors considered highest-risk.

### 2.3. Circulating Tumor Cell Analysis

No patients reported adverse events or complications from blood collection. Serial peripheral venous blood draws were collected; the first (baseline) sample was collected after primary tumor diagnosis or diagnosis of metastatic disease. We used one 10 mL tube of blood containing CellSaveTM preservative for the detection of CTCs using the CellSearch Circulating Melanoma Cell Assay^®^. Circulating tumor cell assessments were performed within 72 h of blood collection as per the manufacturer’s protocol. The CellSearch^®^ Circulating Melanoma Cell test uses ferrofluids coated with CD146 antibodies to immunomagnetically enrich melanoma cells, label the nuclei of these cells with the fluorescent dye 4,2-diamidino-2-phenylindole dihydrochloride (DAPI), and stain them using fluorescently labeled antibodies to detect the combination of high molecular weight melanoma-associated antigen (HMW-MAA; clone 9.2.27), CD45, and CD34. A semi-automated fluorescence-based microscope system was employed to identify circulating tumor cells CD146+, HMW-MAA+, CD45−, CD34−, and nucleated (DAPI+) cells.

### 2.4. Statistical Analysis

We used *t*-tests to compare the mean number of circulating tumor cells between early-stage and metastatic uveal melanoma. Landmark overall survival was calculated using the landest “R” package. Kaplan–Meier curves were derived using the ‘survival’ package in R, for comparison of landmark overall survival between groups of patients stratified by the presence of one or more baseline circulating tumor cell. The *p*-values were two-tailed and values <0.05 were considered statistically significant.

## 3. Results

The median age of the data set was 52 years (20–83 years) and all patients were non-Hispanic Caucasians. Forty-four percent (17/39) of the patients were male and 56% (22/39) female. At the time of study enrollment, 51% (20/39) had early-stage disease and 49% (19/39) had metastatic disease. For early-stage disease, results of gene expression profiling (Decision-DX assay) were available for 75% (15/20) of the patients; 87% (13/15) were Class 2 and 13% (2/15) were Class 1. Mutational analysis data was present for 19 of 20 patients (49%) out of which 12 (63%) had a GNAQ mutation, 5 (26%) had a GNA11 mutation, one (5%) patient was wild-type for both genes and one (5%) had another mutation separate from GNAQ or GNA11. The median time from diagnosis to blood sampling to checking for CTC for the whole cohort was 20.35 months (20.10 months for early-stage vs. 24.65 months for metastatic disease). At the time of data cutoff, 1 June 2018, the median study follow up for the whole cohort was 16.4 months (16.84 months for early-stage vs. 14.56 months for metastatic disease) (Table 1).

### 3.1. CTCs Are More Frequently Detected in Metastatic Uveal Melanoma Compared to Early-Stage Uveal Melanoma

**Figure 1:**
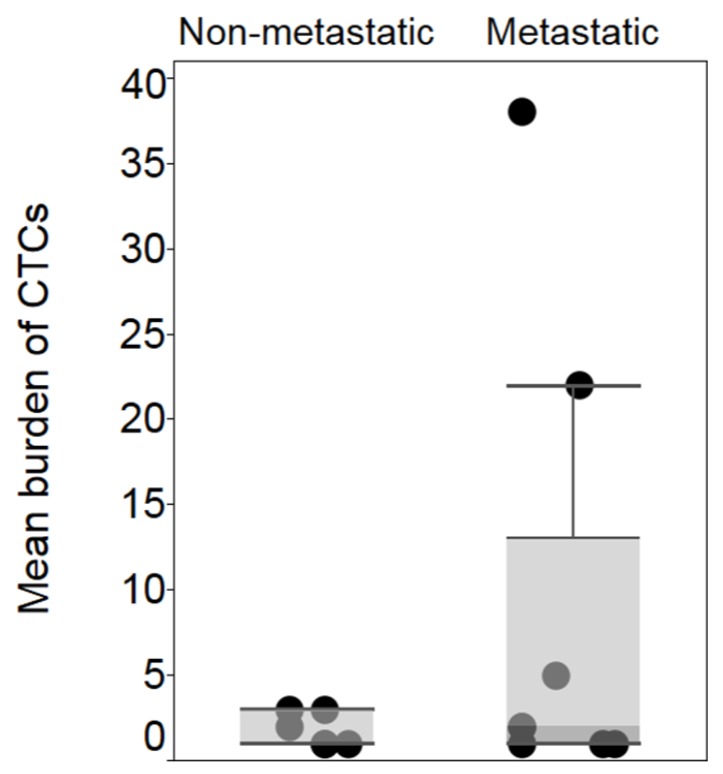
Mean burden of CTC in early-stage, 1.83 CTCs (standard deviation: 0.98), vs. metastatic uveal melanoma 9 CTCs (standard deviation: 13.7). *p* > 0.05.

At initial blood draw, at least one CTC was detected in 36% (14/39) of the patients in the whole cohort, 30% (6/20) of the early-stage disease group, and 42% (8/19) of the patients in the metastatic group (Table 2). Of the eight patients for whom ≥1 CTC was detected at initial blood draw in the metastatic group, three patients had one CTC detected, two patients had two CTCs, and one patient had five, twenty-two and thirty-eight CTCs respectively. In the early-stage disease group, of the six patients who had CTCs detected at initial draw, three had one CTC, one had two CTCs and two had three CTCs. At the median follow up of 16.4 months, the rate of detection of CTCs increased to 54% (21/39), 13 (68.4%) patients in metastatic group had ≥1 CTC detected vs. eight (40%) in the early-stage disease group. Three patients (two patients in the metastatic group and one in the early-stage group) with detectable CTC at initial draw had no detectable CTC during subsequent draws in the study follow-up. The 21 patients included those three patients as well. Out of the eight patients with early-stage disease who had ≥1 CTC detected, five had Class 2 status, one had Class 1A status, and two had an unknown gene expression profile. The mean number of CTCs was higher for the metastatic group compared to the early-stage group, nine (1–38) vs. 1.83 (1–3) respectively. (*p*-value = 0.184) (Figure 1).

### 3.2. Presence of CTC Predicts Metastasis Risk in Early-Stage Uveal Melanoma

**Figure 2:**
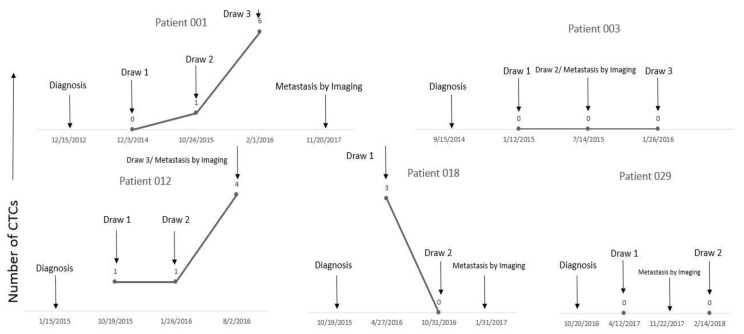
Three out of five patients who developed distant metastasis had CTCs detected before radiographic detection of metastasis. Patients 001, 012, and 018 had CTCs detected 24.9, 9.5 and 3.2 months prior to radiographic evidence of metastasis, respectively. Patient 003 and 029 were “non-secretors” for CTCs as they had no CTC detected pre and post metastasis detection by radiographic imaging.

Metastatic disease developed in five out of 20 (25%) patients in the early-stage group. Eighty percent of these (4/5) were Class 2 and 20% (1/5) had unknown gene expression profiles; however, this patient was known to harbor a monosomy 3 karyotype, a well-established risk factor. In three out of five patients (60%), CTCs were detected before metastatic disease was detected by radiographic imaging. Patients 001, 012, and 018 had CTCs detected 24.9 months, 9.5 months and 3.2 months, respectively, before radiographic detection of distant metastasis. (Table 3, Figure 2). 

### 3.3. CTC Detection Is Risk Factor for Increased Mortality in Early-Stage Uveal Melanoma

**Figure 3:**
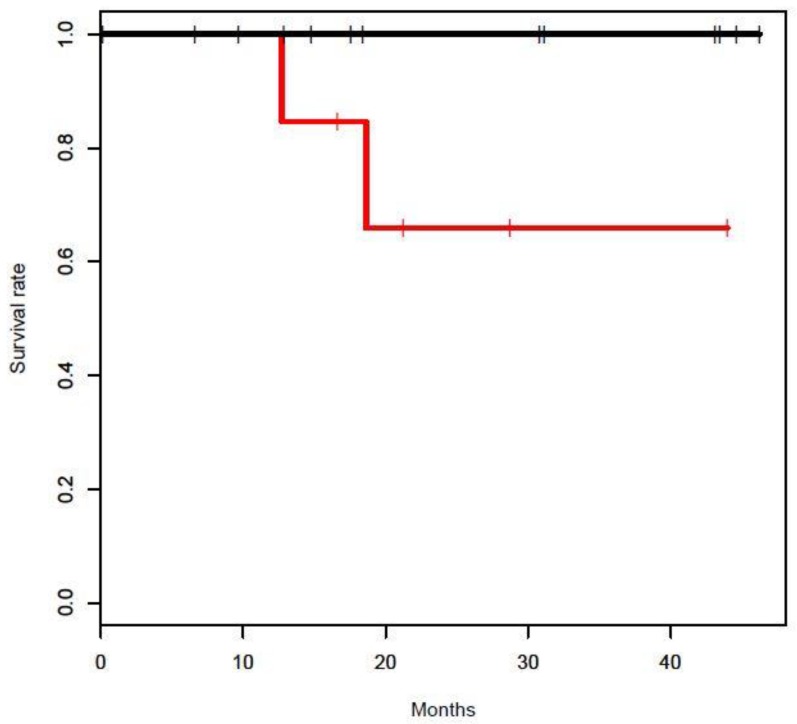
Landmark overall survival at 24 months was poor in CTC detected vs. CTC not-detected early-stage uveal melanoma (*p* < 0.05).

At study cutoff two patients in the early-stage group had died, secondary to disease progression. One patient was Class 2 and the other was unknown with monosomy 3. Both patients had detectable CTCs at initial draw. Landmark OS of CTC positive vs. negative was compared for early-stage uveal melanoma at 12, 24, and 36 months; the *p*-value at 12 months was not applicable, at 24 months was 0.047 and at 36 months was *p* = 0.051 (Figure 3). Nine of 19 (47%) patients in the metastatic group had died at study cutoff. Of the 9 patients, six patients had detectable CTC at either initial draw (4) or subsequent draw (2).

## 4. Discussion

This study was a pilot project conducted with the primary aim to check feasibility of CTC detection in uveal melanoma patients. At initial draw, 36% (14/39) of the patients had detectable CTCs, which increased to 54% (21/39) during course of the study follow-up. CTCs were most frequently detected amongst the metastatic group compared to the early-stage disease group. Our study, along with others [10,11,12,13], confirms that CTCs are detectable in primary uveal melanoma. In early-stage disease, the presence of CTCs correlated with gene expression profiling. Of the patients with ≥1 CTC, 75% (6/8) had gene expression profiling and 83% (5/6) had Class 2 disease. In the early-stage disease group, three patients (60%) had detectable CTCs in the blood before detection of distant metastasis by radiological imaging (MRI) and two patients who did not have any CTC present before detection of distant metastasis by imaging remained negative for CTC presence for at least one more draw. Non-detection of CTCs was limited by the timing of the blood draw in relation to CTC shedding or biological factors [14]. We speculated that there were two groups of early-stage uveal melanoma patients—“CTC secretors” and “CTC non-secretors”. Nonetheless, our study along with the studies by Schuster et al. [13] and Keilholz et al. [15] show the importance of CTC detection in predicting early metastasis in uveal melanoma. In our study comparing early-stage CTC detected vs. non detected uveal melanoma, landmark overall survival at 24 months was statistically low (*p* < 0.05). The reason for not reaching statistical difference at 36 months was likely the small cohort of patients, one of the study limitations. The IRB-approved protocol is ongoing in order to increase accrual, and to permit sequential circulating tumor cell measurements during routine follow-up visits. Measurement of circulating tumor DNA (ctDNA) is another important tool in liquid biopsy to monitor disease status of uveal melanoma. A study by Beasley et al. [12] showed that ctDNA was able to detect metastatic disease earlier than imaging while Bidard et al. showed that in metastatic uveal melanoma ctDNA predicted poor outcomes [16]. Liquid biopsy remains an active area of interest in regards to uveal melanoma. CTCs can even be tested for high-risk cytogenetics. Tura et al. correlated the presence of monosomy 3 in CTCs with its presence in primary tumors [17]. Additional studies coupling CTC detection with other strategies such as ctDNA and single-cell sequencing [18] may provide better complementary diagnostic and prognostic information compared to traditional radiographic surveillance. There remains a need to incorporate liquid biopsy in clinical trials evaluating adjuvant therapy post primary tumor treatment in early-stage uveal melanoma to indicate patients at high-risk of distant metastasis.

## 5. Conclusions

Circulating tumor cells are more frequently detected in the metastatic stage compared to in early-stage uveal melanoma. In early-stage uveal melanoma, most patients in whom circulating tumor cells were detected had adverse prognostic risk factors (Class 2 status by gene expression profiling and monosomy 3). The presence of circulating tumor cells in early-stage disease predicted increased risk of distant metastasis and worse clinical outcomes. The clinical utility of circulating tumor cells in uveal melanoma is not yet known; however, in early-stage disease, longitudinal tracking of circulating tumor cells may serve as a surrogate or complementary tool to radiographic imaging for the detection of microscopic residual disease. In metastatic uveal melanoma, they could also be tracked alongside radiographic scans to determine disease burden and/or response to therapy, ultimately serving as a less costly tool for more efficient assessment than traditional diagnostic imaging. Further studies of early-stage and metastatic uveal melanoma are needed before recommending the routine use of circulating tumor cells in clinical practice.

## Figures and Tables

**Table 1:** Patient characteristics

Total Patients (*n*) = 39	
Sex	
Male (*n*)	17 (44%)
Female (*n*)	22 (56%)
Median age	52 years (20–83 years)
Race	
Non-Hispanic White (*n*)	39 (100%)
Disease status at study enrollment	
Early-stage disease (*n*)	20 (51%)
Class 1 (*n*)	2
Class 2 (*n*)	13
Unknown (*n*)	5
Metastatic (*n*)	19 (49%)
Mutation analysis (*n*)	19
GNAQ (*n*)	12 (63%)
GNA11 (*n*)	5 (27%)
Wildtype (*n*)	1 (5%)
Other (*n*)	1 (5%)
Median time between diagnosis and blood sampling	20.35 months
Early-stage	20.10 months
Metastatic	24.65 months
Total Study follow up	16.40 months
Early-stage	16.84 months
Metastatic	14.56 months

**Table 2:** Circulating tumor cells at initial draw

Early-Stage Uveal Melanoma (*n*)	20
**No CTC detected (*n*)**	14 (70%)
**CTC detected ≥ 1 (*n*)**	14 (70%)
1 CTC	3
Class 1	1
Class 2	2
Unknown	0
2 CTCs	1
Class 1	0
Class 2	0
Unknown	1
3 CTCs	2
Class 1	0
Class 2	1
Unknown	1
**Metastatic Uveal Melanoma (*n*)**	**19**
**No CTC detected (*n*)**	11 (58%)
**CTC detected ≥ 1 (*n*)**	8 (42%)
1 CTC	3
2 CTCs	2
5 CTCs	1
22 CTCs	1
38 CTCs	1

**Table 3:** Characteristics of patients who developed metastasis in early-stage uveal melanoma

Patient	Class by Gene Expression	Mutational Analysis	Date of Diagnosis	Date of CTC Detection	Date of Metastasis by Imaging	Vital Status at Study Cutoff
Patient 001	Class 2	GNA11	12/15/2012	12/3/2014	11/20/2017	Alive
Patient 003	Class 2	*GNAQ*	9/15/2014	CTC never detected	7/14/2015	Alive
Patient 012	Class 2	*GNAQ*	1/15/2015	10/19/2015	8/2/2016	Deceased
Patient 018	Unknown *	wild-type	10/19/2015	4/27/2016	1/31/2017	Deceased
Patient 029	Class 2	not tested	10/20/2016	CTC never detected	11/22/2017	Alive

* monosomy 3. Data: month/day/year.
